# Tetrakis[μ-3-(3-hy­droxy­phen­yl)propenoato]bis­{aqua­(2,2′-bipyridine)­[3-(3-hy­droxy­phen­yl)propenoato]neodymium(III)} 2,2′-bipyridine disolvate dihydrate

**DOI:** 10.1107/S1600536811050707

**Published:** 2011-11-30

**Authors:** Jing-Ke Guo, Yi-Hang Wen

**Affiliations:** a Zhejiang Key Laboratory for Reactive Chemistry on Solid Surfaces, College of Chemistry and Life Science, Zhejiang Normal University, Jinhua, Zhejiang 321004, People’s Republic of China

## Abstract

The dinuclear title compound, [Nd_2_(C_9_H_7_O_3_)_6_(C_10_H_8_N_2_)_2_]·2C_10_H_8_N_2_·2H_2_O, was synthesized under hydro­thermal conditions. The centrosymmetric complex consists of two nine-coordinated Nd^3+^ cations, six 3-hy­droxy­cinnamate anions and two chelating 2,2′-bipyridine mol­ecules. The coordination geometry around the cations can be best described as distorted tricapped trigonal-prismatic. The carboxyl­ate groups show different coordination and bridging modes. Two of them chelate to one Nd^3+^ cation, two bridge the two cations in a bis-monodentate fashion, and two chelate to one and bridge monodentately to the symmetry-related Nd^3+^ cation. The dinuclear mol­ecule is surrounded by two 2,2′-bipyridine solvent and two water mol­ecules. Extensive O—H⋯O and O—H⋯N hydrogen-bonding inter­actions between the components lead to the formation of a three-dimensional network.

## Related literature

For related structures, see: Casas *et al.* (2008 ▶); Crowther *et al.* (2008 ▶); Gossauer *et al.* (2004 ▶); Zhang *et al.* (2010 ▶).
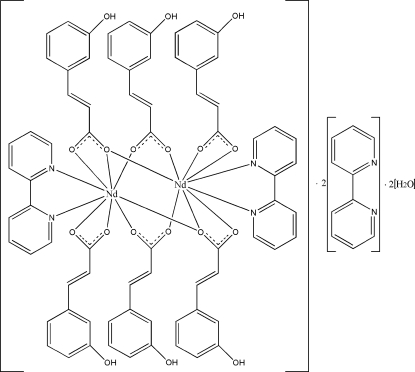


## Experimental

### 

#### Crystal data

[Nd_2_(C_9_H_7_O_3_)_6_(C_10_H_8_N_2_)_2_]·2C_10_H_8_N_2_·2H_2_O
*M* *_r_* = 1928.12
Monoclinic, 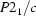

*a* = 10.7333 (2) Å
*b* = 28.9077 (5) Å
*c* = 14.3276 (3) Å
β = 108.274 (1)°
*V* = 4221.30 (14) Å^3^
*Z* = 2
Mo *K*α radiation
μ = 1.30 mm^−1^
*T* = 296 K
0.24 × 0.11 × 0.07 mm

#### Data collection

Bruker APEXII area-detector diffractometer
Absorption correction: multi-scan (*SADABS*; Sheldrick, 1996 ▶) *T*_min_ = 0.84, *T*_max_ = 0.91
36893 measured reflections
9735 independent reflections
6140 reflections with *I* > 2σ(*I*)
*R*_int_ = 0.078

#### Refinement

*R*[*F* ^2^ > 2σ(*F* ^2^)] = 0.045
*wR*(*F* ^2^) = 0.083
*S* = 1.00
9735 reflections
574 parameters
7 restraints
H atoms treated by a mixture of independent and constrained refinement
Δρ_max_ = 1.13 e Å^−3^
Δρ_min_ = −0.65 e Å^−3^

### 

Data collection: *APEX2* (Bruker, 2006 ▶); cell refinement: *SAINT* (Bruker, 2006 ▶); data reduction: *SAINT*; program(s) used to solve structure: *SHELXS97* (Sheldrick, 2008 ▶); program(s) used to refine structure: *SHELXL97* (Sheldrick, 2008 ▶); molecular graphics: *DIAMOND* (Brandenburg, 1999 ▶); software used to prepare material for publication: *SHELXTL* (Sheldrick, 2008 ▶).

## Supplementary Material

Crystal structure: contains datablock(s) I, global. DOI: 10.1107/S1600536811050707/wm2552sup1.cif

Structure factors: contains datablock(s) I. DOI: 10.1107/S1600536811050707/wm2552Isup2.hkl

Additional supplementary materials:  crystallographic information; 3D view; checkCIF report

## Figures and Tables

**Table 1:** Hydrogen-bond geometry (Å, °)

*D*—H⋯*A*	*D*—H	H⋯*A*	*D*⋯*A*	*D*—H⋯*A*
O1*W*—H1*WB*⋯N4^i^	0.87 (5)	2.04 (5)	2.897 (6)	168 (5)
O3—H3⋯O1*W*^ii^	0.96 (4)	1.68 (2)	2.618 (5)	163 (5)
O6—H6⋯O1^iii^	0.93 (4)	1.79 (2)	2.703 (4)	168 (4)
O9—H9⋯N3^i^	0.96 (4)	1.90 (2)	2.849 (5)	175 (5)
O1*W*—H1*WA*⋯O8	0.87 (4)	1.96 (4)	2.825 (4)	175 (5)

Symmetry codes: (i) 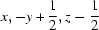; (ii) 
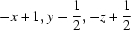
; (iii) 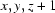.

**Fig. 1.**
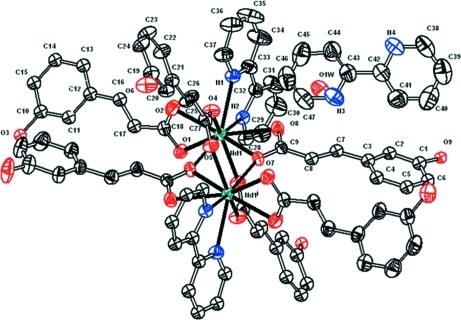
The molecular structure of title compound. Displacement ellipsoids are drawn at the 30% probability level. [Symmetry code: (i) -x + 2,-y,-z + 1]

**Fig. 2.**
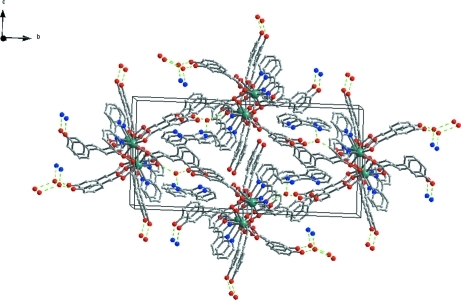
View of the supramolecular network defined by hydrogen bonding interactions.

## supplementary crystallographic information

### Comment

Compounds containing metal ions with 3-hydroxycinnamate ligands (*L*) have been reported previously (e.g. Casas *et al.*, 2008; Crowther *et al.*, 2008; Gossauer *et al.*, 2004; Zhang *et al.*, 2010). Herein we report a new Nd^3+^ compound, Nd_2_*L*_6_(bipy)_2_^.^2(bipy)^.^2(H_2_O), derived from 3-hydroxycinnamic acid and 2,2'-bipyridine (bipy) ligands.

A perspective view of the molecular struture of the centrosymmetric binuclear compound is presented in Fig. 1. It contains two nine-coordinated Nd^3+^ cations, which are linked by four carboxylate groups from four 3-hydroxycinnamate anions, and are also coordinated by two N atoms from two chelating 2,2'-bipyridine molecules. The molecule is surrounded by two solvent 2,2'-bipyridine and two solvent H_2_O molecules. The carboxylate groups adopt different coordination and bridging modes. Two groups are chelating; two are monodentate and bridging; two are both chelating and bridging. Corresponding Nd—O distances are in the range 2.380 (3) to 2.635 (2) Å, with an Nd···Nd separation of 3.9928 (2) Å. Two N atoms of 2,2'-bipyridine [Nd—N distances are 2.617 (3) and 2.646 (3) Å] complete the nine-coordinate configuration of Nd^3+^. Its coordination geometry can be best described as a distorted tricapped trigonal prism.

The dihedral angles between two pyridyl rings from the coordinating and the solvent 2,2'-bipyridine molecules are quite different (10.65 (13) and 48.61 (16) °, respectively). There are extensive intermolecular O—H···N and O—H···O hydrogen-bonding interactions involving the 3-hydroxycinnamate anions, the solvent 2,2'-bipyridine and water molecules (Table 1), resulting in the formation of three-dimensional network structure (Fig. 2).

### Experimental

A mixture of Nd(NO_3_)_3_ (0.3302 g, 0.5 mmol) ,3-hydroxycinnamic acid (0.2462 g, 1.5 mmol) and 2,2'-bipyridine (0.2343 g, 1.5 mmol) was dissolved in 16 mL EtOH/H_2_O (*v*/*v*, 1:15) and then sealed in a 25 ml stainless steel reactor with a telflon liner and heated at 433 K for 72 h, and subsequently cooled to room temperature over 3 days. Then, the reactor was cooled to room temperature at a speed of 5 Kh^-1^. Colourless single crystals of the title compound were obtained by slow evaporation of the filtrate over a few days.

### Refinement

The carbon-bound H-atoms were positioned geometrically and included in the refinement using a riding model [C—H 0.93Å *U*_iso_(H) = 1.2*U*_eq_(C)]. Water H atoms were located in different maps and refined with distance restraints of O—H = 0.85 (2) Å and H—H = 1.35 Å, with displacement parameters set at 1.5*U*_eq_(O).

### Crystal data

**Table:**

[Nd_2_(C_9_H_7_O_3_)_6_(C_10_H_8_N_2_)_2_]·2C_10_H_8_N_2_·2H_2_O	*F*(000) = 1956
*M**_r_* = 1928.12	*D*_x_ = 1.517 Mg m^−^^3^
Monoclinic, *P*2_1_/*c*	Mo *K*α radiation, λ = 0.71073 Å
Hall symbol: -P 2ybc	Cell parameters from 3747 reflections
*a* = 10.7333 (2) Å	θ = 1.4–27.6°
*b* = 28.9077 (5) Å	µ = 1.30 mm^−^^1^
*c* = 14.3276 (3) Å	*T* = 296 K
β = 108.274 (1)°	Block, colorless
*V* = 4221.30 (14) Å^3^	0.24 × 0.11 × 0.07 mm
*Z* = 2	

### Data collection

**Table:**

Bruker APEXII area-detector diffractometer	9735 independent reflections
Radiation source: fine-focus sealed tube	6140 reflections with *I* > 2σ(*I*)
graphite	*R*_int_ = 0.078
ω scans	θ_max_ = 27.6°, θ_min_ = 1.4°
Absorption correction: multi-scan (*SADABS*; Sheldrick, 1996)	*h* = −13→13
*T*_min_ = 0.84, *T*_max_ = 0.91	*k* = −31→37
36893 measured reflections	*l* = −12→18

### Refinement

**Table:**

Refinement on *F*^2^	Primary atom site location: structure-invariant direct methods
Least-squares matrix: full	Secondary atom site location: difference Fourier map
*R*[*F*^2^ > 2σ(*F*^2^)] = 0.045	Hydrogen site location: inferred from neighbouring sites
*wR*(*F*^2^) = 0.083	H atoms treated by a mixture of independent and constrained refinement
*S* = 1.00	*w* = 1/[σ^2^(*F*_o_^2^) + (0.0225*P*)^2^] where *P* = (*F*_o_^2^ + 2*F*_c_^2^)/3
9735 reflections	(Δ/σ)_max_ = 0.001
574 parameters	Δρ_max_ = 1.13 e Å^−^^3^
7 restraints	Δρ_min_ = −0.65 e Å^−^^3^

### Special details

**Table:**

Geometry. All e.s.d.'s (except the e.s.d. in the dihedral angle between two l.s. planes) are estimated using the full covariance matrix. The cell e.s.d.'s are taken into account individually in the estimation of e.s.d.'s in distances, angles and torsion angles; correlations between e.s.d.'s in cell parameters are only used when they are defined by crystal symmetry. An approximate (isotropic) treatment of cell e.s.d.'s is used for estimating e.s.d.'s involving l.s. planes.
Refinement. Refinement of *F*^2^ against ALL reflections. The weighted *R*-factor *wR* and goodness of fit *S* are based on *F*^2^, conventional *R*-factors *R* are based on *F*, with *F* set to zero for negative *F*^2^. The threshold expression of *F*^2^ > σ(*F*^2^) is used only for calculating *R*-factors(gt) *etc*. and is not relevant to the choice of reflections for refinement. *R*-factors based on *F*^2^ are statistically about twice as large as those based on *F*, and *R*- factors based on ALL data will be even larger.

### Fractional atomic coordinates and isotropic or equivalent isotropic displacement parameters (Å^2^)

**Table:**

	*x*	*y*	*z*	*U*_iso_*/*U*_eq_	
Nd1	0.831518 (18)	0.015918 (7)	0.397023 (15)	0.02703 (7)	
N1	0.6264 (3)	0.07010 (11)	0.3784 (2)	0.0384 (8)	
N2	0.7276 (3)	0.06032 (11)	0.2317 (2)	0.0341 (8)	
N3	1.0336 (4)	0.19224 (15)	0.6936 (3)	0.0627 (11)	
N4	0.9520 (4)	0.30801 (15)	0.6957 (3)	0.0653 (12)	
O1	0.7649 (2)	−0.04827 (9)	0.27042 (19)	0.0390 (7)	
O1W	0.8072 (4)	0.18385 (12)	0.3349 (3)	0.0835 (12)	
H1WA	0.845 (5)	0.1579 (12)	0.358 (4)	0.125*	
H1WB	0.841 (5)	0.1884 (19)	0.288 (3)	0.125*	
O2	0.6300 (3)	−0.03295 (10)	0.3539 (2)	0.0475 (8)	
O3	0.3636 (3)	−0.25709 (12)	0.1387 (3)	0.0891 (13)	
H3	0.290 (3)	−0.2761 (15)	0.138 (4)	0.107*	
O4	0.8074 (2)	0.01453 (9)	0.55653 (19)	0.0407 (7)	
O5	0.9911 (2)	−0.00966 (9)	0.67152 (18)	0.0362 (7)	
O6	0.8780 (3)	−0.06697 (11)	1.1306 (2)	0.0553 (9)	
H6	0.837 (4)	−0.0650 (14)	1.179 (2)	0.066*	
O7	1.0478 (2)	0.05061 (8)	0.52033 (18)	0.0313 (6)	
O8	0.9189 (2)	0.09865 (8)	0.4161 (2)	0.0389 (7)	
O9	1.2759 (3)	0.30599 (12)	0.3496 (3)	0.0791 (11)	
H9	1.196 (3)	0.3049 (17)	0.296 (3)	0.095*	
C1	1.2941 (5)	0.27173 (16)	0.4170 (4)	0.0547 (13)	
C2	1.2018 (4)	0.23699 (13)	0.4114 (3)	0.0436 (11)	
H2A	1.1224	0.2376	0.3608	0.052*	
C3	1.2264 (4)	0.20148 (13)	0.4801 (3)	0.0376 (10)	
C4	1.3427 (4)	0.20126 (15)	0.5574 (3)	0.0461 (11)	
H4A	1.3602	0.1778	0.6041	0.055*	
C5	1.4324 (5)	0.23641 (17)	0.5640 (4)	0.0648 (15)	
H5A	1.5093	0.2370	0.6169	0.078*	
C6	1.4101 (5)	0.27068 (17)	0.4937 (4)	0.0640 (15)	
H6A	1.4737	0.2932	0.4980	0.077*	
C7	1.1278 (4)	0.16482 (14)	0.4648 (3)	0.0395 (10)	
H7A	1.0478	0.1713	0.4177	0.047*	
C8	1.1346 (4)	0.12442 (13)	0.5070 (3)	0.0375 (10)	
H8A	1.2111	0.1168	0.5568	0.045*	
C9	1.0275 (4)	0.09037 (13)	0.4796 (3)	0.0322 (9)	
C10	0.3132 (5)	−0.21370 (16)	0.1416 (4)	0.0548 (13)	
C11	0.4023 (4)	−0.17935 (15)	0.1817 (3)	0.0472 (12)	
H11A	0.4917	−0.1857	0.2021	0.057*	
C12	0.3574 (4)	−0.13491 (15)	0.1914 (3)	0.0407 (10)	
C13	0.2243 (4)	−0.12596 (16)	0.1601 (3)	0.0529 (12)	
H13A	0.1942	−0.0965	0.1678	0.063*	
C14	0.1352 (5)	−0.16042 (18)	0.1175 (4)	0.0609 (14)	
H14A	0.0459	−0.1539	0.0947	0.073*	
C15	0.1795 (4)	−0.20420 (18)	0.1091 (3)	0.0616 (14)	
H15A	0.1199	−0.2276	0.0815	0.074*	
C16	0.4520 (4)	−0.09847 (14)	0.2386 (3)	0.0440 (11)	
H16A	0.4213	−0.0750	0.2701	0.053*	
C17	0.5764 (4)	−0.09551 (13)	0.2408 (3)	0.0393 (10)	
H17A	0.6112	−0.1183	0.2105	0.047*	
C18	0.6605 (4)	−0.05660 (13)	0.2908 (3)	0.0337 (9)	
C19	0.7990 (4)	−0.05164 (14)	1.0418 (3)	0.0391 (10)	
C20	0.8559 (4)	−0.04089 (14)	0.9707 (3)	0.0385 (10)	
H20A	0.9461	−0.0443	0.9849	0.046*	
C21	0.7824 (4)	−0.02509 (13)	0.8783 (3)	0.0338 (10)	
C22	0.6472 (4)	−0.01986 (14)	0.8582 (3)	0.0421 (11)	
H22A	0.5954	−0.0096	0.7967	0.050*	
C23	0.5911 (4)	−0.03002 (15)	0.9300 (3)	0.0500 (12)	
H23A	0.5014	−0.0256	0.9171	0.060*	
C24	0.6654 (4)	−0.04660 (15)	1.0208 (3)	0.0455 (11)	
H24A	0.6253	−0.0543	1.0676	0.055*	
C25	0.8479 (4)	−0.01611 (13)	0.8042 (3)	0.0358 (9)	
H25A	0.9390	−0.0175	0.8263	0.043*	
C26	0.7942 (4)	−0.00642 (13)	0.7111 (3)	0.0371 (10)	
H26A	0.7035	−0.0034	0.6871	0.045*	
C27	0.8703 (4)	0.00003 (13)	0.6416 (3)	0.0334 (10)	
C28	0.7741 (4)	0.05261 (14)	0.1567 (3)	0.0401 (10)	
H28A	0.8311	0.0279	0.1615	0.048*	
C29	0.7424 (4)	0.07910 (16)	0.0735 (3)	0.0563 (13)	
H29A	0.7740	0.0716	0.0219	0.068*	
C30	0.6637 (5)	0.11667 (18)	0.0676 (4)	0.0704 (16)	
H30A	0.6431	0.1361	0.0132	0.084*	
C31	0.6153 (5)	0.12521 (16)	0.1442 (4)	0.0640 (15)	
H31A	0.5615	0.1507	0.1416	0.077*	
C32	0.6458 (4)	0.09633 (14)	0.2249 (3)	0.0393 (10)	
C33	0.5880 (4)	0.10092 (15)	0.3063 (3)	0.0433 (11)	
C34	0.4948 (5)	0.13386 (18)	0.3052 (4)	0.0732 (16)	
H34A	0.4721	0.1562	0.2560	0.088*	
C35	0.4354 (5)	0.1334 (2)	0.3779 (4)	0.088 (2)	
H35A	0.3727	0.1555	0.3783	0.106*	
C36	0.4694 (5)	0.10040 (19)	0.4486 (4)	0.0692 (15)	
H36A	0.4280	0.0986	0.4966	0.083*	
C37	0.5658 (4)	0.07005 (16)	0.4474 (3)	0.0507 (12)	
H37A	0.5910	0.0480	0.4971	0.061*	
C38	1.0081 (6)	0.3500 (2)	0.7036 (5)	0.0863 (19)	
H38A	0.9706	0.3743	0.7280	0.104*	
C39	1.1184 (7)	0.3586 (2)	0.6771 (5)	0.095 (2)	
H39A	1.1532	0.3883	0.6820	0.114*	
C40	1.1758 (6)	0.3233 (3)	0.6436 (5)	0.097 (2)	
H40A	1.2495	0.3285	0.6241	0.117*	
C41	1.1234 (5)	0.2798 (2)	0.6391 (4)	0.0735 (16)	
H41A	1.1633	0.2548	0.6191	0.088*	
C42	1.0109 (5)	0.27347 (16)	0.6644 (3)	0.0523 (12)	
C43	0.9506 (5)	0.22716 (17)	0.6563 (3)	0.0536 (13)	
C44	0.8195 (5)	0.22010 (18)	0.6091 (4)	0.0713 (16)	
H44A	0.7640	0.2452	0.5868	0.086*	
C45	0.7705 (6)	0.1761 (2)	0.5949 (5)	0.0888 (19)	
H45A	0.6821	0.1710	0.5618	0.107*	
C46	0.8526 (7)	0.1401 (2)	0.6297 (5)	0.0864 (19)	
H46A	0.8219	0.1099	0.6197	0.104*	
C47	0.9818 (7)	0.14907 (19)	0.6802 (4)	0.0776 (17)	
H47A	1.0366	0.1242	0.7065	0.093*	

### Atomic displacement parameters (Å^2^)

**Table:**

	*U*^11^	*U*^22^	*U*^33^	*U*^12^	*U*^13^	*U*^23^
Nd1	0.02956 (11)	0.02835 (12)	0.02408 (12)	−0.00029 (10)	0.00971 (9)	0.00308 (11)
N1	0.0387 (19)	0.041 (2)	0.037 (2)	0.0041 (15)	0.0140 (18)	0.0011 (18)
N2	0.0314 (17)	0.041 (2)	0.028 (2)	0.0013 (14)	0.0063 (16)	0.0028 (16)
N3	0.078 (3)	0.057 (3)	0.052 (3)	0.014 (2)	0.020 (2)	−0.004 (2)
N4	0.075 (3)	0.051 (3)	0.069 (3)	0.016 (2)	0.020 (3)	−0.002 (2)
O1	0.0421 (16)	0.0423 (17)	0.0365 (18)	−0.0090 (13)	0.0178 (14)	−0.0054 (14)
O1W	0.079 (3)	0.048 (2)	0.130 (4)	0.0187 (18)	0.042 (3)	0.019 (2)
O2	0.0482 (17)	0.0483 (19)	0.054 (2)	−0.0147 (13)	0.0275 (16)	−0.0184 (16)
O3	0.070 (2)	0.057 (3)	0.142 (4)	−0.0180 (18)	0.036 (3)	−0.022 (3)
O4	0.0400 (16)	0.0577 (19)	0.0272 (16)	0.0100 (14)	0.0144 (14)	0.0092 (15)
O5	0.0337 (15)	0.0500 (18)	0.0273 (16)	0.0039 (13)	0.0131 (13)	0.0050 (14)
O6	0.0544 (19)	0.086 (2)	0.0281 (18)	0.0196 (17)	0.0169 (16)	0.0153 (18)
O7	0.0415 (12)	0.0225 (15)	0.0278 (15)	−0.0023 (11)	0.0078 (10)	0.0015 (12)
O8	0.0383 (16)	0.0288 (16)	0.0443 (19)	0.0016 (12)	0.0052 (15)	0.0083 (14)
O9	0.081 (3)	0.062 (2)	0.081 (3)	−0.034 (2)	0.007 (2)	0.029 (2)
C1	0.059 (3)	0.048 (3)	0.058 (4)	−0.017 (2)	0.019 (3)	0.003 (3)
C2	0.046 (3)	0.037 (3)	0.045 (3)	−0.011 (2)	0.011 (2)	−0.001 (2)
C3	0.040 (2)	0.032 (2)	0.041 (3)	−0.0062 (18)	0.014 (2)	−0.003 (2)
C4	0.043 (3)	0.044 (3)	0.048 (3)	−0.007 (2)	0.010 (2)	0.001 (2)
C5	0.049 (3)	0.072 (4)	0.066 (4)	−0.013 (3)	0.008 (3)	0.000 (3)
C6	0.061 (3)	0.062 (4)	0.066 (4)	−0.032 (3)	0.014 (3)	−0.004 (3)
C7	0.035 (2)	0.042 (3)	0.038 (3)	−0.0013 (19)	0.007 (2)	0.003 (2)
C8	0.036 (2)	0.033 (3)	0.039 (3)	−0.0012 (18)	0.006 (2)	0.006 (2)
C9	0.039 (2)	0.027 (2)	0.033 (3)	0.0019 (18)	0.014 (2)	0.000 (2)
C10	0.058 (3)	0.041 (3)	0.069 (4)	−0.007 (2)	0.025 (3)	−0.008 (3)
C11	0.044 (3)	0.044 (3)	0.051 (3)	−0.012 (2)	0.011 (2)	−0.007 (2)
C12	0.044 (2)	0.045 (3)	0.033 (3)	−0.012 (2)	0.013 (2)	−0.002 (2)
C13	0.053 (3)	0.051 (3)	0.054 (3)	−0.006 (2)	0.016 (3)	−0.002 (3)
C14	0.051 (3)	0.066 (4)	0.061 (4)	−0.011 (3)	0.009 (3)	−0.003 (3)
C15	0.056 (3)	0.066 (4)	0.058 (4)	−0.026 (3)	0.012 (3)	−0.013 (3)
C16	0.047 (3)	0.043 (3)	0.041 (3)	−0.007 (2)	0.012 (2)	−0.005 (2)
C17	0.050 (3)	0.033 (3)	0.036 (3)	−0.0044 (19)	0.014 (2)	−0.003 (2)
C18	0.041 (2)	0.030 (2)	0.027 (2)	−0.0016 (18)	0.008 (2)	0.001 (2)
C19	0.041 (2)	0.048 (3)	0.029 (3)	0.002 (2)	0.012 (2)	−0.001 (2)
C20	0.034 (2)	0.048 (3)	0.036 (3)	0.0041 (19)	0.014 (2)	−0.002 (2)
C21	0.038 (2)	0.038 (3)	0.027 (2)	0.0010 (17)	0.013 (2)	−0.0041 (19)
C22	0.037 (2)	0.062 (3)	0.029 (2)	0.000 (2)	0.013 (2)	0.002 (2)
C23	0.035 (2)	0.076 (4)	0.041 (3)	−0.002 (2)	0.016 (2)	0.000 (3)
C24	0.044 (3)	0.068 (3)	0.034 (3)	−0.007 (2)	0.026 (2)	0.005 (2)
C25	0.033 (2)	0.046 (3)	0.032 (2)	0.0017 (19)	0.016 (2)	0.002 (2)
C26	0.034 (2)	0.050 (3)	0.030 (2)	0.0030 (18)	0.015 (2)	0.006 (2)
C27	0.042 (2)	0.031 (2)	0.028 (2)	0.0016 (17)	0.012 (2)	−0.0014 (19)
C28	0.038 (2)	0.048 (3)	0.035 (3)	0.0058 (19)	0.012 (2)	0.005 (2)
C29	0.062 (3)	0.069 (4)	0.042 (3)	0.002 (3)	0.022 (3)	0.012 (3)
C30	0.094 (4)	0.073 (4)	0.045 (3)	0.028 (3)	0.023 (3)	0.033 (3)
C31	0.082 (4)	0.053 (3)	0.057 (4)	0.030 (3)	0.022 (3)	0.024 (3)
C32	0.038 (2)	0.035 (3)	0.040 (3)	0.0043 (19)	0.007 (2)	0.006 (2)
C33	0.041 (2)	0.041 (3)	0.044 (3)	0.009 (2)	0.007 (2)	−0.001 (2)
C34	0.083 (4)	0.084 (4)	0.053 (4)	0.048 (3)	0.022 (3)	0.020 (3)
C35	0.087 (4)	0.110 (5)	0.073 (4)	0.061 (4)	0.033 (4)	0.008 (4)
C36	0.057 (3)	0.097 (4)	0.058 (4)	0.026 (3)	0.025 (3)	−0.001 (3)
C37	0.043 (3)	0.067 (3)	0.042 (3)	0.011 (2)	0.013 (2)	0.006 (3)
C38	0.108 (5)	0.049 (4)	0.097 (5)	0.016 (3)	0.025 (4)	0.007 (4)
C39	0.120 (6)	0.066 (5)	0.092 (5)	−0.013 (4)	0.023 (5)	0.011 (4)
C40	0.101 (5)	0.106 (6)	0.093 (5)	−0.040 (4)	0.043 (4)	−0.018 (4)
C41	0.074 (4)	0.078 (4)	0.074 (4)	−0.003 (3)	0.030 (3)	−0.026 (3)
C42	0.064 (3)	0.049 (3)	0.041 (3)	0.004 (2)	0.012 (3)	0.001 (2)
C43	0.068 (3)	0.050 (3)	0.045 (3)	0.000 (3)	0.020 (3)	−0.003 (3)
C44	0.065 (4)	0.062 (4)	0.076 (4)	−0.003 (3)	0.008 (3)	0.002 (3)
C45	0.082 (4)	0.085 (5)	0.090 (5)	−0.028 (4)	0.014 (4)	−0.006 (4)
C46	0.120 (6)	0.063 (4)	0.081 (5)	−0.021 (4)	0.038 (5)	−0.013 (4)
C47	0.121 (5)	0.053 (4)	0.064 (4)	0.019 (4)	0.037 (4)	0.004 (3)

### Geometric parameters (Å, °)

**Table:**

Nd1—O4	2.380 (3)	C14—C15	1.370 (6)
Nd1—O5^i^	2.407 (2)	C14—H14A	0.9300
Nd1—O7^i^	2.412 (2)	C15—H15A	0.9300
Nd1—O2	2.494 (2)	C16—C17	1.328 (5)
Nd1—O1	2.536 (3)	C16—H16A	0.9300
Nd1—O8	2.552 (2)	C17—C18	1.479 (5)
Nd1—N2	2.617 (3)	C17—H17A	0.9300
Nd1—O7	2.635 (2)	C19—C20	1.378 (5)
Nd1—N1	2.646 (3)	C19—C24	1.379 (5)
N1—C33	1.329 (5)	C20—C21	1.388 (5)
N1—C37	1.343 (5)	C20—H20A	0.9300
N2—C28	1.337 (5)	C21—C22	1.396 (5)
N2—C32	1.346 (4)	C21—C25	1.469 (5)
N3—C43	1.342 (5)	C22—C23	1.377 (5)
N3—C47	1.355 (6)	C22—H22A	0.9300
N4—C42	1.333 (5)	C23—C24	1.381 (5)
N4—C38	1.343 (6)	C23—H23A	0.9300
O1—C18	1.267 (4)	C24—H24A	0.9300
O1W—H1WA	0.87 (4)	C25—C26	1.307 (5)
O1W—H1WB	0.87 (19)	C25—H25A	0.9300
O2—C18	1.256 (4)	C26—C27	1.484 (5)
O3—C10	1.372 (5)	C26—H26A	0.9300
O3—H3	0.96 (4)	C28—C29	1.368 (5)
O4—C27	1.266 (4)	C28—H28A	0.9300
O5—C27	1.262 (4)	C29—C30	1.362 (6)
O5—Nd1^i^	2.407 (2)	C29—H29A	0.9300
O6—C19	1.363 (4)	C30—C31	1.376 (6)
O6—H6	0.93 (4)	C30—H30A	0.9300
O7—C9	1.277 (4)	C31—C32	1.379 (6)
O7—Nd1^i^	2.412 (2)	C31—H31A	0.9300
O8—C9	1.256 (4)	C32—C33	1.488 (6)
O9—C1	1.354 (5)	C33—C34	1.378 (5)
O9—H9	0.96 (4)	C34—C35	1.381 (7)
C1—C6	1.378 (6)	C34—H34A	0.9300
C1—C2	1.395 (5)	C35—C36	1.356 (7)
C2—C3	1.389 (5)	C35—H35A	0.9300
C2—H2A	0.9300	C36—C37	1.361 (5)
C3—C4	1.384 (5)	C36—H36A	0.9300
C3—C7	1.465 (5)	C37—H37A	0.9300
C4—C5	1.383 (5)	C38—C39	1.375 (8)
C4—H4A	0.9300	C38—H38A	0.9300
C5—C6	1.379 (6)	C39—C40	1.354 (7)
C5—H5A	0.9300	C39—H39A	0.9300
C6—H6A	0.9300	C40—C41	1.372 (7)
C7—C8	1.306 (5)	C40—H40A	0.9300
C7—H7A	0.9300	C41—C42	1.377 (6)
C8—C9	1.470 (5)	C41—H41A	0.9300
C8—H8A	0.9300	C42—C43	1.476 (6)
C10—C11	1.373 (5)	C43—C44	1.372 (6)
C10—C15	1.390 (6)	C44—C45	1.366 (6)
C11—C12	1.394 (5)	C44—H44A	0.9300
C11—H11A	0.9300	C45—C46	1.353 (7)
C12—C13	1.381 (5)	C45—H45A	0.9300
C12—C16	1.472 (5)	C46—C47	1.373 (7)
C13—C14	1.383 (6)	C46—H46A	0.9300
C13—H13A	0.9300	C47—H47A	0.9300
			
O4—Nd1—O5^i^	136.78 (8)	O3—C10—C15	123.1 (4)
O4—Nd1—O7^i^	73.65 (8)	C11—C10—C15	120.5 (4)
O5^i^—Nd1—O7^i^	76.62 (8)	C10—C11—C12	119.4 (4)
O4—Nd1—O2	83.19 (9)	C10—C11—H11A	120.3
O5^i^—Nd1—O2	126.29 (9)	C12—C11—H11A	120.3
O7^i^—Nd1—O2	87.75 (9)	C13—C12—C11	119.6 (4)
O4—Nd1—O1	125.69 (9)	C13—C12—C16	120.6 (4)
O5^i^—Nd1—O1	74.62 (8)	C11—C12—C16	119.7 (4)
O7^i^—Nd1—O1	75.84 (8)	C12—C13—C14	120.7 (4)
O2—Nd1—O1	51.69 (8)	C12—C13—H13A	119.7
O4—Nd1—O8	93.43 (9)	C14—C13—H13A	119.7
O5^i^—Nd1—O8	78.64 (8)	C15—C14—C13	119.5 (5)
O7^i^—Nd1—O8	124.88 (8)	C15—C14—H14A	120.2
O2—Nd1—O8	144.94 (9)	C13—C14—H14A	120.2
O1—Nd1—O8	140.72 (8)	C14—C15—C10	120.2 (4)
O4—Nd1—N2	137.21 (9)	C14—C15—H15A	119.9
O5^i^—Nd1—N2	80.48 (9)	C10—C15—H15A	119.9
O7^i^—Nd1—N2	147.84 (9)	C17—C16—C12	126.9 (4)
O2—Nd1—N2	87.88 (9)	C17—C16—H16A	116.5
O1—Nd1—N2	76.52 (9)	C12—C16—H16A	116.5
O8—Nd1—N2	71.06 (9)	C16—C17—C18	121.2 (4)
O4—Nd1—O7	72.59 (8)	C16—C17—H17A	119.4
O5^i^—Nd1—O7	70.06 (8)	C18—C17—H17A	119.4
O7^i^—Nd1—O7	75.50 (8)	O2—C18—O1	120.8 (4)
O2—Nd1—O7	153.53 (9)	O2—C18—C17	120.3 (4)
O1—Nd1—O7	138.55 (8)	O1—C18—C17	118.9 (4)
O8—Nd1—O7	49.80 (8)	O2—C18—Nd1	59.42 (19)
N2—Nd1—O7	117.13 (8)	O1—C18—Nd1	61.38 (19)
O4—Nd1—N1	76.64 (9)	C17—C18—Nd1	176.9 (3)
O5^i^—Nd1—N1	137.98 (9)	O6—C19—C20	118.3 (4)
O7^i^—Nd1—N1	145.40 (9)	O6—C19—C24	122.5 (4)
O2—Nd1—N1	71.38 (10)	C20—C19—C24	119.2 (4)
O1—Nd1—N1	108.79 (9)	C19—C20—C21	121.8 (4)
O8—Nd1—N1	73.88 (9)	C19—C20—H20A	119.1
N2—Nd1—N1	60.90 (10)	C21—C20—H20A	119.1
O7—Nd1—N1	111.86 (9)	C20—C21—C22	118.5 (4)
O4—Nd1—C18	104.56 (10)	C20—C21—C25	119.3 (3)
O5^i^—Nd1—C18	100.60 (10)	C22—C21—C25	122.2 (4)
O7^i^—Nd1—C18	80.48 (9)	C23—C22—C21	119.4 (4)
O2—Nd1—C18	25.69 (9)	C23—C22—H22A	120.3
O1—Nd1—C18	26.01 (9)	C21—C22—H22A	120.3
O8—Nd1—C18	152.73 (10)	C22—C23—C24	121.3 (4)
N2—Nd1—C18	81.88 (10)	C22—C23—H23A	119.3
O7—Nd1—C18	155.64 (9)	C24—C23—H23A	119.3
N1—Nd1—C18	90.31 (10)	C19—C24—C23	119.7 (4)
O4—Nd1—C9	84.66 (10)	C19—C24—H24A	120.1
O5^i^—Nd1—C9	70.51 (9)	C23—C24—H24A	120.1
O7^i^—Nd1—C9	100.21 (10)	C26—C25—C21	128.2 (4)
O2—Nd1—C9	162.98 (9)	C26—C25—H25A	115.9
O1—Nd1—C9	144.81 (9)	C21—C25—H25A	115.9
O8—Nd1—C9	24.69 (8)	C25—C26—C27	123.5 (4)
N2—Nd1—C9	93.00 (10)	C25—C26—H26A	118.2
O7—Nd1—C9	25.31 (8)	C27—C26—H26A	118.2
N1—Nd1—C9	94.23 (10)	O5—C27—O4	125.6 (4)
C18—Nd1—C9	170.47 (11)	O5—C27—C26	117.8 (4)
O4—Nd1—Nd1^i^	68.44 (6)	O4—C27—C26	116.6 (3)
O5^i^—Nd1—Nd1^i^	68.60 (6)	N2—C28—C29	123.6 (4)
O7^i^—Nd1—Nd1^i^	39.71 (6)	N2—C28—H28A	118.2
O2—Nd1—Nd1^i^	124.54 (7)	C29—C28—H28A	118.2
O1—Nd1—Nd1^i^	110.29 (6)	C30—C29—C28	118.7 (4)
O8—Nd1—Nd1^i^	85.38 (6)	C30—C29—H29A	120.7
N2—Nd1—Nd1^i^	144.33 (6)	C28—C29—H29A	120.7
O7—Nd1—Nd1^i^	35.79 (5)	C29—C30—C31	118.4 (5)
N1—Nd1—Nd1^i^	137.98 (7)	C29—C30—H30A	120.8
C18—Nd1—Nd1^i^	120.09 (8)	C31—C30—H30A	120.8
C9—Nd1—Nd1^i^	60.69 (8)	C30—C31—C32	120.7 (4)
C33—N1—C37	118.0 (4)	C30—C31—H31A	119.7
C33—N1—Nd1	121.3 (3)	C32—C31—H31A	119.7
C37—N1—Nd1	120.4 (3)	N2—C32—C31	120.4 (4)
C28—N2—C32	118.1 (4)	N2—C32—C33	115.8 (4)
C28—N2—Nd1	119.0 (3)	C31—C32—C33	123.7 (4)
C32—N2—Nd1	121.9 (3)	N1—C33—C34	121.3 (4)
C43—N3—C47	116.4 (5)	N1—C33—C32	116.5 (3)
C42—N4—C38	117.2 (5)	C34—C33—C32	122.2 (4)
C18—O1—Nd1	92.6 (2)	C33—C34—C35	119.4 (5)
H1WA—O1W—H1WB	100 (3)	C33—C34—H34A	120.3
C18—O2—Nd1	94.9 (2)	C35—C34—H34A	120.3
C10—O3—H3	101 (3)	C36—C35—C34	119.4 (5)
C27—O4—Nd1	138.1 (2)	C36—C35—H35A	120.3
C27—O5—Nd1^i^	137.5 (2)	C34—C35—H35A	120.3
C19—O6—H6	112 (3)	C35—C36—C37	118.1 (5)
C9—O7—Nd1^i^	158.6 (2)	C35—C36—H36A	120.9
C9—O7—Nd1	92.7 (2)	C37—C36—H36A	120.9
Nd1^i^—O7—Nd1	104.50 (8)	N1—C37—C36	123.7 (4)
C9—O8—Nd1	97.2 (2)	N1—C37—H37A	118.2
C1—O9—H9	116 (3)	C36—C37—H37A	118.2
O9—C1—C6	118.5 (4)	N4—C38—C39	122.8 (6)
O9—C1—C2	122.9 (4)	N4—C38—H38A	118.6
C6—C1—C2	118.6 (4)	C39—C38—H38A	118.6
C3—C2—C1	121.1 (4)	C40—C39—C38	119.3 (6)
C3—C2—H2A	119.4	C40—C39—H39A	120.4
C1—C2—H2A	119.4	C38—C39—H39A	120.4
C4—C3—C2	119.6 (4)	C39—C40—C41	118.8 (6)
C4—C3—C7	123.0 (4)	C39—C40—H40A	120.6
C2—C3—C7	117.4 (4)	C41—C40—H40A	120.6
C5—C4—C3	119.0 (4)	C40—C41—C42	119.3 (5)
C5—C4—H4A	120.5	C40—C41—H41A	120.3
C3—C4—H4A	120.5	C42—C41—H41A	120.3
C6—C5—C4	121.4 (5)	N4—C42—C41	122.5 (5)
C6—C5—H5A	119.3	N4—C42—C43	117.6 (4)
C4—C5—H5A	119.3	C41—C42—C43	119.9 (5)
C1—C6—C5	120.2 (4)	N3—C43—C44	122.3 (5)
C1—C6—H6A	119.9	N3—C43—C42	115.5 (5)
C5—C6—H6A	119.9	C44—C43—C42	122.0 (5)
C8—C7—C3	130.1 (4)	C45—C44—C43	119.9 (5)
C8—C7—H7A	114.9	C45—C44—H44A	120.0
C3—C7—H7A	114.9	C43—C44—H44A	120.0
C7—C8—C9	123.3 (4)	C46—C45—C44	119.0 (6)
C7—C8—H8A	118.3	C46—C45—H45A	120.5
C9—C8—H8A	118.3	C44—C45—H45A	120.5
O8—C9—O7	119.3 (3)	C45—C46—C47	118.9 (6)
O8—C9—C8	122.4 (4)	C45—C46—H46A	120.6
O7—C9—C8	118.3 (4)	C47—C46—H46A	120.6
O8—C9—Nd1	58.09 (19)	N3—C47—C46	123.4 (5)
O7—C9—Nd1	61.94 (19)	N3—C47—H47A	118.3
C8—C9—Nd1	169.8 (3)	C46—C47—H47A	118.3
O3—C10—C11	116.4 (4)		
			
O4—Nd1—N1—C33	159.5 (3)	O5^i^—Nd1—C9—O8	105.6 (2)
O5^i^—Nd1—N1—C33	10.7 (4)	O7^i^—Nd1—C9—O8	177.4 (2)
O7^i^—Nd1—N1—C33	−169.1 (3)	O2—Nd1—C9—O8	−65.7 (4)
O2—Nd1—N1—C33	−113.3 (3)	O1—Nd1—C9—O8	97.5 (2)
O1—Nd1—N1—C33	−76.9 (3)	N2—Nd1—C9—O8	26.8 (2)
O8—Nd1—N1—C33	61.9 (3)	O7—Nd1—C9—O8	−170.0 (4)
N2—Nd1—N1—C33	−15.1 (3)	N1—Nd1—C9—O8	−34.2 (2)
O7—Nd1—N1—C33	94.8 (3)	Nd1^i^—Nd1—C9—O8	−178.6 (2)
C18—Nd1—N1—C33	−95.6 (3)	O4—Nd1—C9—O7	59.7 (2)
C9—Nd1—N1—C33	76.0 (3)	O5^i^—Nd1—C9—O7	−84.4 (2)
Nd1^i^—Nd1—N1—C33	125.4 (3)	O7^i^—Nd1—C9—O7	−12.6 (2)
O4—Nd1—N1—C37	−14.3 (3)	O2—Nd1—C9—O7	104.2 (4)
O5^i^—Nd1—N1—C37	−163.1 (3)	O1—Nd1—C9—O7	−92.5 (2)
O7^i^—Nd1—N1—C37	17.1 (4)	O8—Nd1—C9—O7	170.0 (4)
O2—Nd1—N1—C37	73.0 (3)	N2—Nd1—C9—O7	−163.2 (2)
O1—Nd1—N1—C37	109.3 (3)	N1—Nd1—C9—O7	135.8 (2)
O8—Nd1—N1—C37	−111.9 (3)	Nd1^i^—Nd1—C9—O7	−8.57 (17)
N2—Nd1—N1—C37	171.2 (3)	O4—Nd1—C9—C8	153.6 (16)
O7—Nd1—N1—C37	−79.0 (3)	O5^i^—Nd1—C9—C8	9.5 (16)
C18—Nd1—N1—C37	90.6 (3)	O7^i^—Nd1—C9—C8	81.3 (16)
C9—Nd1—N1—C37	−97.7 (3)	O2—Nd1—C9—C8	−161.8 (14)
Nd1^i^—Nd1—N1—C37	−48.4 (3)	O1—Nd1—C9—C8	1.4 (17)
O4—Nd1—N2—C28	176.6 (2)	O8—Nd1—C9—C8	−96.1 (16)
O5^i^—Nd1—N2—C28	21.6 (3)	N2—Nd1—C9—C8	−69.3 (16)
O7^i^—Nd1—N2—C28	−23.4 (4)	O7—Nd1—C9—C8	93.9 (16)
O2—Nd1—N2—C28	−105.8 (3)	N1—Nd1—C9—C8	−130.3 (16)
O1—Nd1—N2—C28	−54.7 (3)	Nd1^i^—Nd1—C9—C8	85.4 (16)
O8—Nd1—N2—C28	102.8 (3)	O3—C10—C11—C12	−176.8 (4)
O7—Nd1—N2—C28	83.3 (3)	C15—C10—C11—C12	1.3 (7)
N1—Nd1—N2—C28	−175.6 (3)	C10—C11—C12—C13	−0.4 (7)
C18—Nd1—N2—C28	−80.7 (3)	C10—C11—C12—C16	177.1 (4)
C9—Nd1—N2—C28	91.3 (3)	C11—C12—C13—C14	−1.3 (7)
Nd1^i^—Nd1—N2—C28	51.4 (3)	C16—C12—C13—C14	−178.8 (4)
O4—Nd1—N2—C32	8.6 (3)	C12—C13—C14—C15	2.0 (7)
O5^i^—Nd1—N2—C32	−146.4 (3)	C13—C14—C15—C10	−1.1 (8)
O7^i^—Nd1—N2—C32	168.6 (2)	O3—C10—C15—C14	177.4 (5)
O2—Nd1—N2—C32	86.2 (3)	C11—C10—C15—C14	−0.5 (8)
O1—Nd1—N2—C32	137.2 (3)	C13—C12—C16—C17	−154.3 (5)
O8—Nd1—N2—C32	−65.3 (3)	C11—C12—C16—C17	28.3 (7)
O7—Nd1—N2—C32	−84.8 (3)	C12—C16—C17—C18	179.7 (4)
N1—Nd1—N2—C32	16.4 (3)	Nd1—O2—C18—O1	−2.1 (4)
C18—Nd1—N2—C32	111.3 (3)	Nd1—O2—C18—C17	176.4 (3)
C9—Nd1—N2—C32	−76.8 (3)	Nd1—O1—C18—O2	2.0 (4)
Nd1^i^—Nd1—N2—C32	−116.6 (3)	Nd1—O1—C18—C17	−176.4 (3)
O4—Nd1—O1—C18	40.0 (3)	C16—C17—C18—O2	19.7 (6)
O5^i^—Nd1—O1—C18	177.1 (2)	C16—C17—C18—O1	−161.9 (4)
O7^i^—Nd1—O1—C18	97.4 (2)	O4—Nd1—C18—O2	34.7 (2)
O2—Nd1—O1—C18	−1.1 (2)	O5^i^—Nd1—C18—O2	179.2 (2)
O8—Nd1—O1—C18	−134.1 (2)	O7^i^—Nd1—C18—O2	104.9 (2)
N2—Nd1—O1—C18	−99.2 (2)	O1—Nd1—C18—O2	−178.0 (4)
O7—Nd1—O1—C18	144.9 (2)	O8—Nd1—C18—O2	−95.1 (3)
N1—Nd1—O1—C18	−46.8 (2)	N2—Nd1—C18—O2	−102.1 (2)
C9—Nd1—O1—C18	−175.0 (2)	O7—Nd1—C18—O2	114.5 (3)
Nd1^i^—Nd1—O1—C18	117.4 (2)	N1—Nd1—C18—O2	−41.6 (2)
O4—Nd1—O2—C18	−146.3 (2)	Nd1^i^—Nd1—C18—O2	107.9 (2)
O5^i^—Nd1—O2—C18	−1.0 (3)	O4—Nd1—C18—O1	−147.4 (2)
O7^i^—Nd1—O2—C18	−72.5 (2)	O5^i^—Nd1—C18—O1	−2.8 (2)
O1—Nd1—O2—C18	1.1 (2)	O7^i^—Nd1—C18—O1	−77.2 (2)
O8—Nd1—O2—C18	127.4 (2)	O2—Nd1—C18—O1	178.0 (4)
N2—Nd1—O2—C18	75.6 (2)	O8—Nd1—C18—O1	82.9 (3)
O7—Nd1—O2—C18	−122.7 (2)	N2—Nd1—C18—O1	75.8 (2)
N1—Nd1—O2—C18	135.5 (3)	O7—Nd1—C18—O1	−67.5 (3)
C9—Nd1—O2—C18	168.9 (3)	N1—Nd1—C18—O1	136.3 (2)
Nd1^i^—Nd1—O2—C18	−88.4 (2)	Nd1^i^—Nd1—C18—O1	−74.1 (2)
O5^i^—Nd1—O4—C27	−21.2 (4)	O6—C19—C20—C21	180.0 (4)
O7^i^—Nd1—O4—C27	27.2 (4)	C24—C19—C20—C21	0.0 (6)
O2—Nd1—O4—C27	116.8 (4)	C19—C20—C21—C22	0.4 (6)
O1—Nd1—O4—C27	85.5 (4)	C19—C20—C21—C25	−177.4 (4)
O8—Nd1—O4—C27	−98.2 (4)	C20—C21—C22—C23	0.6 (6)
N2—Nd1—O4—C27	−163.8 (3)	C25—C21—C22—C23	178.3 (4)
O7—Nd1—O4—C27	−52.4 (4)	C21—C22—C23—C24	−1.9 (7)
N1—Nd1—O4—C27	−170.8 (4)	O6—C19—C24—C23	178.7 (4)
C18—Nd1—O4—C27	102.5 (4)	C20—C19—C24—C23	−1.3 (7)
C9—Nd1—O4—C27	−75.1 (4)	C22—C23—C24—C19	2.3 (7)
Nd1^i^—Nd1—O4—C27	−14.6 (4)	C20—C21—C25—C26	171.4 (4)
O4—Nd1—O7—C9	−115.8 (2)	C22—C21—C25—C26	−6.3 (7)
O5^i^—Nd1—O7—C9	86.4 (2)	C21—C25—C26—C27	−177.2 (4)
O7^i^—Nd1—O7—C9	167.2 (2)	Nd1^i^—O5—C27—O4	−7.0 (6)
O2—Nd1—O7—C9	−140.5 (2)	Nd1^i^—O5—C27—C26	172.3 (2)
O1—Nd1—O7—C9	119.6 (2)	Nd1—O4—C27—O5	19.2 (6)
O8—Nd1—O7—C9	−5.5 (2)	Nd1—O4—C27—C26	−160.1 (3)
N2—Nd1—O7—C9	18.9 (2)	C25—C26—C27—O5	8.6 (6)
N1—Nd1—O7—C9	−48.5 (2)	C25—C26—C27—O4	−172.0 (4)
C18—Nd1—O7—C9	157.3 (3)	C32—N2—C28—C29	−0.4 (6)
Nd1^i^—Nd1—O7—C9	167.2 (2)	Nd1—N2—C28—C29	−168.9 (3)
O4—Nd1—O7—Nd1^i^	77.07 (10)	N2—C28—C29—C30	2.9 (7)
O5^i^—Nd1—O7—Nd1^i^	−80.77 (9)	C28—C29—C30—C31	−2.5 (8)
O7^i^—Nd1—O7—Nd1^i^	0.0	C29—C30—C31—C32	−0.1 (8)
O2—Nd1—O7—Nd1^i^	52.4 (2)	C28—N2—C32—C31	−2.3 (6)
O1—Nd1—O7—Nd1^i^	−47.59 (15)	Nd1—N2—C32—C31	165.8 (3)
O8—Nd1—O7—Nd1^i^	−172.62 (14)	C28—N2—C32—C33	174.9 (3)
N2—Nd1—O7—Nd1^i^	−148.21 (9)	Nd1—N2—C32—C33	−16.9 (5)
N1—Nd1—O7—Nd1^i^	144.32 (10)	C30—C31—C32—N2	2.6 (7)
C18—Nd1—O7—Nd1^i^	−9.9 (3)	C30—C31—C32—C33	−174.4 (5)
C9—Nd1—O7—Nd1^i^	−167.2 (2)	C37—N1—C33—C34	4.1 (7)
O4—Nd1—O8—C9	69.3 (2)	Nd1—N1—C33—C34	−169.8 (4)
O5^i^—Nd1—O8—C9	−67.8 (2)	C37—N1—C33—C32	−172.8 (4)
O7^i^—Nd1—O8—C9	−3.1 (3)	Nd1—N1—C33—C32	13.2 (5)
O2—Nd1—O8—C9	152.3 (2)	N2—C32—C33—N1	2.2 (6)
O1—Nd1—O8—C9	−115.5 (2)	C31—C32—C33—N1	179.4 (4)
N2—Nd1—O8—C9	−151.5 (2)	N2—C32—C33—C34	−174.8 (4)
O7—Nd1—O8—C9	5.6 (2)	C31—C32—C33—C34	2.4 (7)
N1—Nd1—O8—C9	144.3 (2)	N1—C33—C34—C35	−3.3 (8)
C18—Nd1—O8—C9	−158.9 (3)	C32—C33—C34—C35	173.5 (5)
Nd1^i^—Nd1—O8—C9	1.3 (2)	C33—C34—C35—C36	−0.3 (9)
O9—C1—C2—C3	177.6 (4)	C34—C35—C36—C37	2.8 (9)
C6—C1—C2—C3	−1.3 (7)	C33—N1—C37—C36	−1.5 (7)
C1—C2—C3—C4	2.1 (6)	Nd1—N1—C37—C36	172.5 (4)
C1—C2—C3—C7	−176.0 (4)	C35—C36—C37—N1	−2.0 (8)
C2—C3—C4—C5	−0.4 (6)	C42—N4—C38—C39	2.7 (9)
C7—C3—C4—C5	177.6 (4)	N4—C38—C39—C40	−1.6 (10)
C3—C4—C5—C6	−2.1 (7)	C38—C39—C40—C41	−1.3 (10)
O9—C1—C6—C5	179.9 (5)	C39—C40—C41—C42	2.8 (9)
C2—C1—C6—C5	−1.3 (7)	C38—N4—C42—C41	−1.1 (7)
C4—C5—C6—C1	3.0 (8)	C38—N4—C42—C43	179.5 (5)
C4—C3—C7—C8	−10.7 (7)	C40—C41—C42—N4	−1.7 (8)
C2—C3—C7—C8	167.3 (4)	C40—C41—C42—C43	177.7 (5)
C3—C7—C8—C9	−177.0 (4)	C47—N3—C43—C44	1.5 (7)
Nd1—O8—C9—O7	−10.1 (4)	C47—N3—C43—C42	−175.1 (4)
Nd1—O8—C9—C8	167.9 (3)	N4—C42—C43—N3	−134.0 (5)
Nd1^i^—O7—C9—O8	153.7 (5)	C41—C42—C43—N3	46.5 (6)
Nd1—O7—C9—O8	9.7 (4)	N4—C42—C43—C44	49.3 (7)
Nd1^i^—O7—C9—C8	−24.5 (9)	C41—C42—C43—C44	−130.1 (5)
Nd1—O7—C9—C8	−168.4 (3)	N3—C43—C44—C45	−2.9 (8)
Nd1^i^—O7—C9—Nd1	143.9 (7)	C42—C43—C44—C45	173.5 (5)
C7—C8—C9—O8	−3.1 (6)	C43—C44—C45—C46	1.3 (9)
C7—C8—C9—O7	174.9 (4)	C44—C45—C46—C47	1.4 (10)
C7—C8—C9—Nd1	86.5 (16)	C43—N3—C47—C46	1.4 (8)
O4—Nd1—C9—O8	−110.3 (2)	C45—C46—C47—N3	−2.9 (9)

Symmetry codes: (i) −*x*+2, −*y*, −*z*+1.

### Hydrogen-bond geometry (Å, °)

**Table:**

*D*—H···*A*	*D*—H	H···*A*	*D*···*A*	*D*—H···*A*
O1W—H1WB···N4^ii^	0.87 (5)	2.04 (5)	2.897 (6)	168 (5)
O3—H3···O1W^iii^	0.96 (4)	1.68 (2)	2.618 (5)	163 (5)
O6—H6···O1^iv^	0.93 (4)	1.79 (2)	2.703 (4)	168 (4)
O9—H9···N3^ii^	0.96 (4)	1.90 (2)	2.849 (5)	175 (5)
O1W—H1WA···O8	0.87 (4)	1.96 (4)	2.825 (4)	175 (5)

Symmetry codes: (ii) *x*, −*y*+1/2, *z*−1/2; (iii) −*x*+1, *y*−1/2, −*z*+1/2; (iv) *x*, *y*, *z*+1.
